# Disseminating the Foundations of Knowledge Translation and Patient Engagement Science Through the KnowledgeNudge Blog and Twitter Profile: Quantitative Descriptive Evaluation

**DOI:** 10.2196/15351

**Published:** 2020-06-15

**Authors:** Kathryn M Sibley, Masood Khan, Patricia L Roche, Patrick Faucher, Carly Leggett

**Affiliations:** 1 Department of Community Health Sciences University of Manitoba Winnipeg, MB Canada; 2 George & Fay Yee Centre for Healthcare Innovation Winnipeg, MB Canada

**Keywords:** knowledge translation, dissemination, blog, Twitter

## Abstract

**Background:**

There is a documented need to build capacity for theory- and evidence-informed knowledge translation (KT) and patient engagement (PE) practice in health research. Dissemination of foundational content online coupled with social media promotion may build capacity by increasing awareness, knowledge, and positive attitudes.

**Objective:**

This retrospective study sought to (1) describe exposure and engagement of the KnowledgeNudge KT and PE dissemination strategy (online blog and Twitter profile) over 2 years and (2) identify and compare characteristics of individual posts with the most and least exposure and reach.

**Methods:**

Exposure was assessed by blog site views per month and Twitter profile impressions per month. Engagement was assessed by Twitter profile interactions per month. Descriptive statistics were calculated for 6-month blocks and compared using one-way analysis of variance or Student *t* test. Individual post exposure was assessed by average post views per week. Individual post reach was assessed by average post reads per week. High- and low-profile blog posts with the highest and lowest 10th percentile for exposure and reach were identified.

**Results:**

A total of 99 posts and 755 tweets were published during the study period. There was a significant increase in exposure (*P*=.004) and reach (*P*<.001) during the final 6 months. Seven high-profile and 6 low-profile posts were identified. High-profile posts had a significantly greater average word count than low-profile posts (*P*=.003). There were no other significant differences between posts.

**Conclusions:**

The increases in KnowledgeNudge exposure and engagement offer preliminary evidence in support of this dissemination strategy for the practice of KT and PE. Variation in individual post exposure and reach warrants further exploration to tailor content to user needs. Future work will include a prospective evaluation strategy to explore the effect of KnowledgeNudge on awareness, knowledge, attitudes, and behavior.

## Introduction

Knowledge translation (KT) is an evolving discipline dedicated to advancing the synthesis, exchange, dissemination, and application of knowledge to optimize health, health care delivery, and the health care system [1]. It is a dynamic and iterative process that occurs throughout complex interactions between evidence producers and users [1], including but not limited to people with personal experience of a health issue (as a patient, family member, friend, or caregiver). The conceptual and evidence foundation for KT is growing rapidly, alongside growing recognition of the need for meaningful and active involvement of individuals with personal experience of a health issue in all stages of the research process (known in Canada as patient engagement [PE]) [2].

Accordingly, the need to build capacity to practice theory- and evidence-informed KT and PE has been identified in multiple studies [3-8]. For example, in a 2015 qualitative study of health researchers in Manitoba, Canada, KT education and training resources were identified as most needed to support practicing KT [7]. Similarly, in a 2017 survey of Manitoba health researchers, 81% of respondents indicated the need for training and educational resources to practice PE [9]. While the need for training has been consistently demonstrated, potential solutions need to consider known barriers to participation such as competing priorities and cost, which were identified in a 2014 Canadian study of knowledge translation training needs [10].

Dissemination of foundational resource material may help to build capacity for practicing theory- and evidence-informed KT and PE by facilitating distribution and awareness of seminal works, current best evidence and practices, and ongoing critical debates in the field [8]. In turn, dissemination may lead to increased knowledge and positive attitudes toward theory- and evidence-informed KT and PE, ultimately leading to improved skills and stimulation of behavior change [11]. Although effective dissemination strategies specific to KT and PE content have not been determined, online resources that use web-based technologies have emerged as a popular dissemination strategy due to their potential to reach large numbers of users [12] and have been shown to increase self-reported knowledge, skills, and information exchange in a time- and cost-efficient manner [13,14]. The impact of online dissemination resources may be enhanced when coupled with social media, which is rapidly growing in use in health research and care [15]. A 2015 study of 852 health researchers reported that 26.9% used social media for obtaining research evidence and almost all (95.9%) participants considered social media important for obtaining and disseminating research evidence [16]. Disseminating evidence through social media has also been shown to be effective in stimulating behavior change among health research users. For instance, a 2015 study of 317 clinicians found that 70% of participants reported changing or intending to change their practice after receiving evidence-based practice information through social media [17].

To address the established need to build capacity for practicing evidence-informed KT and PE, the KnowledgeNudge dissemination strategy was developed and launched in 2016. KnowledgeNudge consists of an online blog (a web-based collection of self-published content) [18] and accompanying Twitter profile [19]. The objective of KnowledgeNudge is to increase awareness, knowledge, and positive attitudes of KT and PE and related best practice concepts, considerations, and resources. The ultimate goal of KnowledgeNudge is to influence uptake of evidence-informed and/or best practice behaviors in KT and PE. Target audiences include health researchers, practitioners, and people with lived experience of a health issue. The editorial team comprises an academic KT scientist, KT and PE practice leads, and knowledge brokers with 4 to 10 years of experience. Contributors include a core group of KT scientists, academic trainees, research coordinators, KT and PE practice leads, and knowledge brokers (n=12), as well as guest posts from external health researchers and practitioners, patients/health consumers, and knowledge brokers with related expertise (n=9). Weekly blog posts summarize theoretical concepts, offer practice guidance, and provide opinions on issues of debate. Blog posts are intentionally short (less than 2000 words) and are written in a tone that is engaging, unique, and conversational and uses principles of plain language to appeal to a wide range of readers. Tweets are posted to promote each new blog post. Tweets direct readers to new content on the day of publication and through multiple follow-up posts in the first week. When new posts are not available, tweets promote existing posts.

There is consistent recognition of the need for outcome evaluation in established KT process models [20] and best practice recommendations for online KT tools and dissemination approaches [12]. As an important foundational step in evaluation of the KnowledgeNudge dissemination strategy, the overall goal of this study was to examine spread of KnowledgeNudge during its first 2 years. This concept was operationalized using recommended key performance indicators for social media use in health promotion: exposure, engagement, and reach [21]. Exposure reflects the number of times content has been viewed on a social media application. Engagement has been defined as an indicator of linking social medial to action, at its lowest level indicating some active interaction or participation, with individuals acknowledging agreement or preference for the content [21]. Reach indicates the number of individuals who have contact with the social media application and the related content [21].

The primary objective of this study was to describe and determine changes in exposure and engagement of the KnowledgeNudge blog and Twitter profile over a 2-year period (objective 1). The secondary objective was to identify and compare characteristics of individual posts with the most and least exposure and reach (objective 2). For the purpose of this study, exposure was measured through blog site views and Twitter profile impressions, and engagement was evaluated through Twitter profile interactions. Individual post exposure was evaluated through post views and reach through post reads.

## Methods

### Study Design

A retrospective descriptive quantitative study was conducted from August 1, 2016, through July 31, 2018. Given the nonidentifiable nature of the secondary data used for analysis, research ethics approval was not sought per Article 2.4 of the 2014 Government of Canada Tri-Council Policy Statement on Ethical Conduct for Research Involving Humans [22].

### Data Collection

For objective 1 (describe and determine changes in exposure and engagement of the KnowledgeNudge blog and Twitter profile), study data were extracted from standard use metrics available for the KnowledgeNudge blog site account (hosted on WordPress from August 2016 to February 2017 and on Medium from March 2017 to July 2018) and KnowledgeNudge Twitter profile. For objective 2 (identify and compare characteristics of individual posts with the most and least exposure and reach), metrics for individual posts were collected on October 1, 2018, allowing the last post in the study period (published on July 25, 2018) to accumulate metrics for 68 days. Individual post data were not available for the period August 2016 to February 2017 due to the change in site host and the inability to retrospectively retrieve individual post data from WordPress once the account was closed.

### Outcomes

The primary study outcomes were exposure and engagement of the blog site and Twitter profile (objective 1), as well as individual post exposure and reach (objective 2) [23,24]. Exposure was defined as the total number of KnowledgeNudge blog site page views per month and the total number of Twitter profile impressions per month. Engagement was defined as the total number of Twitter profile engagements per month. Individual post exposure was defined as the average number of views per week, calculated for each post by dividing the total number of post views over the study period by the number of weeks since the post was first published. Individual post reach was defined as the average number of reads per week, calculated for each post by dividing the total number of reads for the study period (a metric provided by Medium, in which a read is counted when someone remains on the post page for the estimated amount of time it takes to read the entire post) by the number of weeks since the post was first published. Secondary outcomes included individual post content characteristics of post topic (knowledge translation, patient engagement, both, or other); post type (conceptual, practice, or other); when each post was published (dividing the study period into 6-month bins); the number of tweets used to share each post during the study period; and the total number of words per post (excluding the title, subtitle, and author information).

### Data Analysis

Descriptive summary statistics were calculated for all variables (values reported are mean and standard deviation). To determine characteristics of individual posts, a combination of deductive and inductive coding was used. First, the research team met and developed a coding framework that consisted of two broad coding categories by topic (KT and PE) and three subcategories by type (practical issues or practice, theoretical concepts or conceptual, opinions, or other). Practice posts were defined as those providing information on tools and resources for KT and PE; conceptual posts were defined as those providing evidence-based information on the science of KT and PE based on peer-reviewed literature; and opinion posts were defined as those providing expert opinion on KT and PE science and practice. Two team members independently coded individual posts. An additional category was included for posts that specifically identified as relevant to both KT and PE, and all other posts that did not fit in the developed categories were coded as other (for example, informative posts about staff members). Coding disagreements were resolved through discussion. Kappa scores were calculated to determine interrater reliability of individual post coding, which was found to be strong for post topic (𝜅=.85) and moderate for post type (𝜅=.66) [25].

To determine changes in exposure and engagement over time (objective 1), a 1-way analysis of variance was conducted to assess average blog site exposure over time and Twitter profile exposure and engagement for each 6-month block. Post hoc comparisons were completed using the Tukey honestly significant difference (HSD) test. Statistical significance was set at *P*<.05.

To identify and compare individual posts with the most and least exposure and reach (objective 2), posts were ranked separately by exposure and reach (average weekly views and reads, respectively). High-profile posts were identified as those in the 10th highest percentile for both exposure and reach, and low-profile posts were identified as those in the 10th lowest percentile for both exposure and reach. Posts with missing data (those originally posted on WordPress and imported to Medium) were included in identification of high-profile posts but not in identifying low-profile posts in order to avoid categorizing these posts as low profile when exposure and reach could not be accurately measured for the entire study period. Tweets per post, word count, and exposure and reach of high- and low-profile posts were compared with a Student *t* test [26]. A 2-sided Fisher exact test was used to compare time of publication (by 6-month block), topic, and type of post for high- and low-profile posts [27].

## Results

### KnowledgeNudge Production Summary

A total of 99 posts were published during the study period (average 4 posts per month). Average post length was 818 words (range 246 to 1908). Of the total posts, 59% (58/99) were characterized as KT, 29% (29/99) as PE, 5% (5/99) as both KT and PE, and 7% (7/99) as other topics such as providing information about the KnowledgeNudge team. Half of the posts (49/99) were coded as practice, 31% (32/99) as conceptual, 12% (12/99) as opinion pieces, and 6% (6/99) as other. A total of 755 tweets were produced during the study period. Most tweets directly promoted individual posts (599/755, 79.3%), while the remainder promoted related content via retweets.

### Objective 1. Exposure and Engagement of KnowledgeNudge

**Figure 1:**
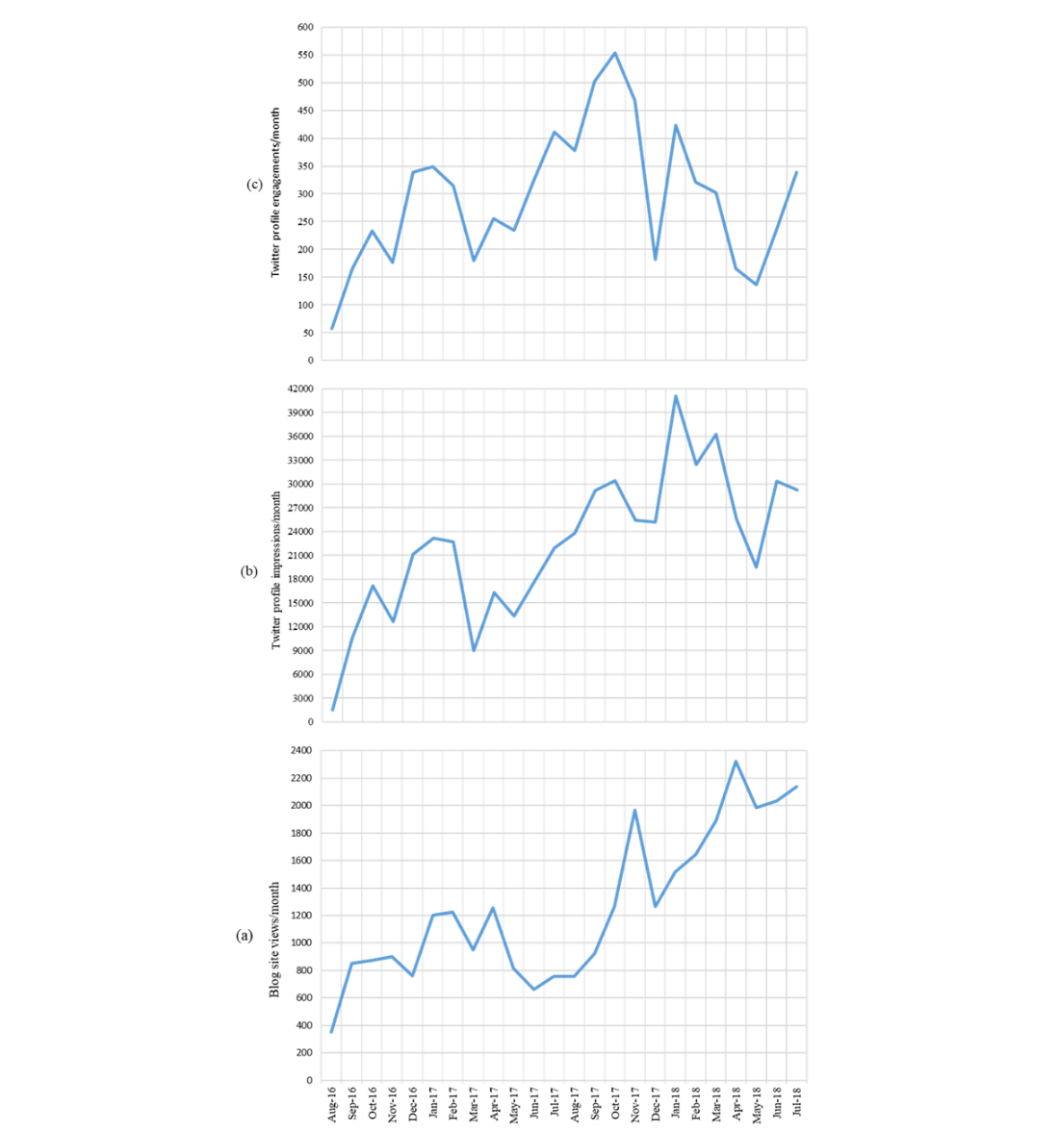
KnowledgeNudge blog site exposure (a), Twitter exposure (b), and Twitter engagement (c) over 2-year period. There was a significant increase in blog site exposure (*P*<.001) and Twitter exposure (*P*=.004) between the first and last 6 months of the study period. No significant difference was seen in Twitter engagement between the first and last 6 months.

Monthly exposure and engagement are displayed in Figure 1. Average blog site exposure was 1263 (SD 549) site views per month during the study period (range 353 to 2322). There was a significant increase in blog site exposure over time (*F*_3,20_=17.9, *P*<.001). Post hoc comparisons using the Tukey HSD test showed that average blog site exposure was significantly greater in the last 6-month block (2002 [SD 229] views per month) than the first (823 [SD 275] views per month) and second (944 [SD 248] views per month) 6-month blocks (*P*=.001) and the third (1283 [SD 432] views per month) 6-month block (*P*=.003). There was no significant difference in average blog site exposure between the first, second, and third 6-month blocks.

Average Twitter exposure was 22,320 (SD 9139) impressions per month (range 1522 to 41,112). A significant increase was seen in exposure over time (*F*_3,20_=9.0, *P*<.001). Post hoc comparison using the Tukey HSD test showed that average Twitter exposure significantly increased from the first 6-month block (14,374 [SD 7903] impressions per month) to the third 6-month block (29,190 [SD 6371] impressions per month; *P*=.003) and to the last 6-month block (28,899 [SD 5780] impressions per month; *P*=.004). Post hoc comparison using the Tukey HSD test also revealed a significant increase in Twitter exposure between the second 6-month block (16,818 impressions per month), third 6-month block (*P*=.02), and last 6-month block (*P*=.02).

Average Twitter engagement was 294 (SD 124) engagements per month (range 57 to 554). There was a significant effect of time on average engagement (*F*_3,20_=4.2, *P*=.02). Post hoc comparison using the Tukey HSD test showed a significant increase in average Twitter engagement between the first 6-month block (220 [SD 119] engagements per month) and the third 6-month block (418 [SD 131] engagements per month; *P*=.02). There were no other significant differences between the 6-month blocks.

### Objective 2: Individual KnowledgeNudge Post Characteristics

The average exposure of individual posts was 4.8 (SD 8.8) views per week (range 0.3 to 79). The average reach of individual posts was 1.7 (SD 1.7) reads per week (range 0.1 to 12). The average word count of individual posts was 988.3 (SD 429.3; range 434 to 1908). The average number of tweets per post was 7.1 (SD 2.3; range 3 to 11). Seven posts were identified as high profile, and 6 posts were identified as low profile. Comparison between high- and low-profile posts using a Student *t* test showed there was a significant difference between high- and low-profile posts for exposure (*t*_1_=2.2, *P*=.02) and reach (*t*_1_=4.9, *P*<.001). The word count of high-profile posts was significantly greater than that of low-profile posts (*t*_1_=3.4, *P*=.003). There was no significant difference in the number of tweets per post between high- and low-profile posts (*t*_1_=1.7, *P*=.06). There was no significant difference between high- and low-profile posts in terms of time of publication (*P*=.66), topic (*P*=.12), or type (*P*=.44). Characteristics of individual high- and low-profile posts are reported in Table 1.

**Table 1:** Characteristics of high- and low-profile KnowledgeNudge posts (those within the highest and lowest 10th percentile for both exposure and reach).

Title	Publication date	Topic	Type	Word count	Tweets/ post	Exposure (views/week)	Reach (reads/week)	Rank
The Knowledge-to-Action Framework	Nov 15, 2017	KT	Conceptual	1908	8	79	12	High
Decolonizing Community Engagement	Nov 22, 2017	PE	Conceptual	1411	11	38	9	High
Infographics: A Primer for Researchers	Jul 25, 2018	KT	Conceptual	1505	5	19	7	High
Knowledge Translation & Translational Research: Are They One & the Same?	Sep 6, 2017	KT	Conceptual	988	6	11	5	High
Budgeting for Patient & Public Engagement in Health Research	Oct 18, 2017	PE	Practice	1142	10	10	4	High
Unpacking KT Theories, Models, and Frameworks	Jan 25, 2017	KT	Conceptual	794	8	9	5	High
Top 10 Knowledge Translation Resources	Jul 12, 2017	KT	Practice	1072	8	9	4	High
Top KnowledgeNudge Posts of 2017	Nov 29, 2017	Other	Other	510	3	0.5	0.3	Low
Photovoice Blog-Series, Blog #2: Community Partnerships & Hard-to-Reach Populations	Aug 16, 2017	PE	Practice	915	4	0.9	0.4	Low
Everything (So Far) in One Post!	Apr 5, 2017	Other	Other	434	7	1	0.4	Low
Networks for Knowledge Translation	May 17, 2017	KT	Conceptual	554	9	1.4	0.8	Low
Sex- and Gender-Based Analysis (SGBA): Importance in Health Research	May 31, 2017	Both	Conceptual	640	7	1.6	0.6	Low
Part-II: The James Lind Alliance: An Overview of the Process of Priority Setting Partnerships	Apr 11, 2018	PE	Conceptual	975	6	1.8	0.7	Low

## Discussion

### Principal Findings

The goal of this study was to explore changes in exposure and engagement of the KnowledgeNudge KT and PE dissemination strategy during its initial 2-year period. By formally measuring and documenting changes in exposure and engagement of the KnowledgeNudge strategy, this study adds to the growing body of literature on the use of social media in dissemination science and demonstrates important contributions for building capacity in the practice of high-quality KT and PE practices. The findings establish a foundation for future studies to explore in-depth the impact of KnowledgeNudge content on KT and PE practice behaviors.

An important finding was the significant increase in blog site exposure between the first and last 6-month periods, providing evidence of increased spread of information on foundational issues of KT and PE. The significant increase in Twitter profile exposure during the study also provides evidence of growing network size. The absence of significant changes in Twitter profile engagement between the first and last 6-month periods is arguably less important, as the Twitter profile served to encourage users to visit the primary blog site and was not the primary source of KT and PE content. We also observed considerable variation in reach among the individual posts. Post topic and type coding did not suggest any trends influencing variation between posts with the highest and lowest reach.

The findings of this study are consistent with published evaluations of exposure and engagement in online dissemination blog strategies and Twitter activity in related domains of health [28-30]. The increase in exposure is a precursor to increase in awareness. The findings also support the goals of KnowledgeNudge by increasing the spread of information on foundational issues relating to the practice of KT and PE. A 2019 scoping review of core competencies for KT identified 19 individual competencies specific to addressing primarily knowledge, skills, or attitudes [31]. Although individual posts were not coded according to this framework, the KnowledgeNudge strategy as a whole seeks to address aspects of each of these competency domains. For example, the KnowledgeNudge dissemination strategy addresses many of these individual competencies related to the domain of knowledge including sharing of knowledge, increasing awareness of evidence resources, and promoting the understanding of research process, KT, and dissemination activities [31]. However, it has yet to be determined whether the KnowledgeNudge dissemination strategy addressed the domains of skills and attitudes. Moving forward, this core competency framework could be used to guide development of KnowledgeNudge content and evaluation approaches.

Although we did not directly assess factors that contributed to the increase in blog site exposure, using Twitter as a promotional strategy has been associated with increase in website traffic [29,32]. For example, a study of a website and Twitter profile aimed at disseminating resources related to child health found that an increase in Twitter exposure was associated with increased website reach [29]. Other factors that might have contributed to the increase in exposure of the blog site include posting content on a regular basis (on average one post per week) and publishing on subject matter identified as being of interest to readers through annual reader surveys. Similarly, having a team of authors with a wide of range of expertise also contributed to producing content covering a vast range of topics in PE and KT, potentially attracting a wide range of readership. Future studies should more explicitly consider and compare the effects of variables such as post frequency and content on changes in exposure, reach, and engagement.

The findings related to characteristics of high- and low-profile posts have important implications for those planning online dissemination strategies in general and those specifically interested in building capacity for KT and PE practices. Among the blog characteristics tested, the only significantly different variable between high- and low-profile posts was word count. Notably, high-profile posts had significantly more words than low-profile posts. This finding is supported by some evidence suggesting longer blogs are more influential in terms of garnering reader attention and feedback [33]. We found no difference in KT or PE post topic, practice or conceptual type, number of tweets per post, or time period of publication between high and low-profile posts. Other metrics not captured in our study may help to explain differences between high- and low-profile posts. The finding that time period of publication was not significantly different does eliminate two important opposite influencing factors: first, that older posts might accumulate more views and reads compared with newer posts because of the former being posted for a longer period on the website, and second, that more recent posts might have higher views and reads compared with older posts because of recency bias [34].

### Limitations

We acknowledge the limitations of this retrospective study and potential for continued evaluation to overcome them. First, we recognize that reliance on online use metrics provides only a proxy indicator of awareness and generally relies on the assumption that the reader is actively interacting with the content. Analysis of related indicators such as Medium comments or claps (Medium’s version of likes) may have provided additional evidence for active engagement and awareness with the KnowledgeNudge strategy, although the inherent difficulty of accessing, measuring, and interpreting social media metrics is recognized in the literature [35]. Retrospective use of standard online metrics also restricted data accessibility to some extent (particularly blog site-level metrics such as unique visitors), and this was compounded by the unanticipated effects of changing host sites during the study period. We recommend those planning similar evaluations ensure in advance that their anticipated metrics are accessible, relevant, and feasible to collect.

Future research opportunities for understanding the effects of the KnowledgeNudge dissemination strategy include (but are not limited to) additional analysis to explore factors influencing variation in individual post engagement (such as author and guest effects; social media influencer effects; tweet content such as hashtags, photos, or language; and timing), social network analysis based on KnowledgeNudge Twitter and/or Medium followers, and a prospective study examining the overall impact of KnowledgeNudge on knowledge, skills, and attitudes toward theory- and evidence-informed KT and PE practice. Recognizing the limitations of dissemination strategies on behavior [36] and given that multifaceted KT strategies have been shown to be more effective [37] the impact of KnowledgeNudge would likely be optimized when coupled with active training and practice-building strategies.

### Conclusions

The KnowledgeNudge dissemination strategy demonstrated increased online blog site and Twitter exposure over an initial 2-year period. Growth of KnowledgeNudge resulted mostly from an increase in blog site and Twitter exposures. The outcomes included in this study, based on standard use data, provide evidence of a foundational component for increasing capacity for theory- and evidence-informed KT and PE practice: access to information. Critical next steps include ongoing evaluation to explore the effects of KnowledgeNudge on knowledge, skills, and behavior. Variation in individual post reach warrants further exploration and audience feedback to tailor content to user needs. Future work will include a prospective evaluation strategy to comprehensively explore the effect of KnowledgeNudge on knowledge, attitudes, and behavior, and opportunities to leverage KnowledgeNudge resources in an active training program will be explored.

## Abbreviations

HSD: honestly significant difference
KT: knowledge translation
PE: patient engagement

## References

[1] (2015). Canadian Institutes of Health Research.

[2] (2019). Canadian Institutes of Health Research.

[3] Eccles MP, Armstrong D, Baker R, Cleary K, Davies H, Davies S, Glasziou P, Ilott I, Kinmonth A, Leng G, Logan S, Marteau T, Michie S, Rogers H, Rycroft-Malone J, Sibbald B (2009). An implementation research agenda. Implement Sci.

[4] Gagliardi AR, Dobrow MJ (2016). Identifying the conditions needed for integrated knowledge translation (IKT) in health care organizations: qualitative interviews with researchers and research users. BMC Health Serv Res.

[5] Harvey G, Marshall RJ, Jordan Z, Kitson AL (2015). Exploring the hidden barriers in knowledge translation: a case study within an academic community. Qual Health Res.

[6] (2012). Michael Smith Foundation for Health Research Vancouver, BC.

[7] Sibley KM, Roche PL, Bell CP, Temple B, Wittmeier KDM (2017). A descriptive qualitative examination of knowledge translation practice among health researchers in Manitoba, Canada. BMC Health Serv Res.

[8] Birdsell JM, Omelchuk K (2007). Alberta Heritage Foundation for Medical Research.

[9] Crockett LK, Shimmin C, Wittmeier KDM, Sibley KM (2019). Engaging patients and the public in health research: experiences, perceptions and training needs among Manitoba health researchers. Res Involv Engagem.

[10] Holmes BJ, Schellenberg M, Schell K, Scarrow G (2014). How funding agencies can support research use in healthcare: an online province-wide survey to determine knowledge translation training needs. Implement Sci.

[11] Lafrenière D, Menuz V, Hurlimann T, Godard B (2013). Knowledge dissemination interventions: a literature review. SAGE Open.

[12] Levac D, Glegg SMN, Camden C, Rivard LM, Missiuna C (2015). Best practice recommendations for the development, implementation, and evaluation of online knowledge translation resources in rehabilitation. Phys Ther.

[13] Curran VR, Fleet L (2005). A review of evaluation outcomes of web-based continuing medical education. Med Educ.

[14] Mairs K, McNeil H, McLeod J, Prorok JC, Stolee P (2013). Online strategies to facilitate health-related knowledge transfer: a systematic search and review. Health Info Libr J.

[15] Harseim T, Goodey G (2017). Of Schemes and Memes.

[16] Tunnecliff J, Ilic D, Morgan P, Keating J, Gaida JE, Clearihan L, Sadasivan S, Davies D, Ganesh S, Mohanty P, Weiner J, Reynolds J, Maloney S (2015). The acceptability among health researchers and clinicians of social media to translate research evidence to clinical practice: mixed-methods survey and interview study. J Med Internet Res.

[17] Maloney S, Tunnecliff J, Morgan P, Gaida JE, Clearihan L, Sadasivan S, Davies D, Ganesh S, Mohanty P, Weiner J, Reynolds J, Ilic D (2015). Translating evidence into practice via social media: a mixed-methods study. J Med Internet Res.

[18] KnowledgeNudge Blog.

[19] KnowledgeNudge Twitter Profile.

[20] Graham ID, Logan J, Harrison MB, Straus SE, Tetroe J, Caswell W, Robinson N (2006). Lost in knowledge translation: time for a map?. J Contin Educ Health Prof.

[21] Neiger BL, Thackeray R, Van Wagenen SA, Hanson CL, West JH, Barnes MD, Fagen MC (2012). Use of social media in health promotion: purposes, key performance indicators, and evaluation metrics. Health Promot Pract.

[22] Canadian Institute of Health Research, Natural Sciences and Engineering Research Council of Canada, Social Sciences and Humanities Research Council of Canada (2014). Tri-Council Policy Statement: Ethical conduct for research involving humans.

[23] About your activity dashboard: what is the Tweet activity dashboard?.

[24] Medium: your stats.

[25] McHugh ML (2012). Interrater reliability: the kappa statistic. Biochem Med (Zagreb).

[26] Kalpic D, Hlupic N, Lovric M, Lovric M (2011). Student's t-tests. International Encyclopedia of Statistical Science.

[27] McDonald JH (2014). Handbook of Biological Statistics, 3rd edition.

[28] Carley S, Beardsell I, May N, Crowe L, Baombe J, Grayson A, Carden R, Liebig A, Gray C, Fisher R, Horner D, Howard L, Body R (2018). Social-media-enabled learning in emergency medicine: a case study of the growth, engagement and impact of a free open access medical education blog. Postgrad Med J.

[29] Gates A, Featherstone R, Shave K, Scott SD, Hartling L (2018). Dissemination of evidence in paediatric emergency medicine: a quantitative descriptive evaluation of a 16-week social media promotion. BMJ Open.

[30] Duke JC, Hansen H, Kim AE, Curry L, Allen J (2014). The use of social media by state tobacco control programs to promote smoking cessation: a cross-sectional study. J Med Internet Res.

[31] Mallidou AA, Atherton P, Chan L, Frisch N, Glegg S, Scarrow G (2018). Core knowledge translation competencies: a scoping review. BMC Health Serv Res.

[32] Dyson MP, Newton AS, Shave K, Featherstone RM, Thomson D, Wingert A, Fernandes RM, Hartling L (2017). Social media for the dissemination of Cochrane Child Health Evidence: evaluation study. J Med Internet Res.

[33] Agarwal N, Liu H, Tang L, Yu P (2008). Identifying the influential bloggers in a community. Proc Int Conf Web Search Web Data Mining.

[34] Murphy J, Hofacker C, Mizerski R (2006). Primacy and recency effects on clicking behavior. J Comp Mediated Comm.

[35] Drula G (2012). Social and online media research: data, metrics and methods.

[36] Gagnon ML (2011). Moving knowledge to action through dissemination and exchange. J Clin Epidemiol.

[37] Grimshaw JM, Shirran L, Thomas R, Mowatt G, Fraser C, Bero L, Grilli R, Harvey E, Oxman A, O'Brien MA (2001). Changing provider behavior: an overview of systematic reviews of interventions. Med Care.

